# IMPACT OF PHYSICAL ACTIVITY CORRELATES IN THE ISOLATED AND COMBINED PRESENCE OF INSUFFICIENT LEVEL OF PHYSICAL ACTIVITY AND HIGH SCREEN TIME AMONG ADOLESCENTS

**DOI:** 10.1590/1984-0462/;2019;37;2;00011

**Published:** 2019-01-21

**Authors:** Thiago Silva Piola, Eliane Denise Araújo Bacil, Michael Pereira Silva, Ana Beatriz Pacífico, Edina Maria de Camargo, Wagner de Campos

**Affiliations:** a Universidade Federal do Paraná, Curitiba, PR, Brazil.

**Keywords:** Motor activity, Sedentary lifestyle, Physical inactivity, Atividade motora, Estilo de vida sedentário, Inatividade física

## Abstract

**Objective:**

To identify the impact of physical activity correlates with the isolated and combined presence of insufficient physical activity and high screen time among adolescents.

**Methods:**

A cross-sectional representative study was carried out with a sample of 786 adolescents (16.0±1.0 years; 53.9% girls) randomly selected in the schools of São José dos Pinhais, Paraná, Southern Brazil. The physical activity correlates analyzed were sex, nutritional status, economic class and sexual maturation. Physical activity level and screen time were measured and classified according to reference criteria. The associations were tested with Poisson regression and the population attributable fraction (PAF) verified the impact of correlates on the combined presence of insufficient level of physical activity and high screen time by the prevalence ratio (PR).

**Results:**

Among the studied adolescents, 84.7% (n=666) were considered insufficiently active, 96.4% (n=758) reported high screen time and 82.1% (n=645) presented the combined presence of these behaviors. The female sex and the high economic status were positively associated with the insufficient level of physical activity (PR=1.19; 95% confidence interval - 95%CI 1.12-1.27; PAF=15.97 - female/adjusted; PR=1,1; 95%CI 1,01-1,19; PAF=9,09 - high/adjusted class). The female sex also was positively associated to high screen time after adjustments (PR=1.18; 95%CI 1.10-1.27; PAF=15.25). The female sex was positively associated with the combined presence of these behaviors (PR=1.18; 95%CI 1.10-1.27) with a 15.25% impact on these behaviors.

**Conclusions:**

Physical activity correlates can have an impact on the insufficient level of physical activity and high screen time, especially among girls.

## RESUMO

**Objetivo:**

Identificar o impacto dos correlatos da atividade física (AF) com a presença isolada e a combinada da prática insuficiente de AFs e do elevado tempo de tela em adolescentes.

**Métodos:**

Estudo transversal, representativo, com amostra de 786 adolescentes (16,2±1,1 anos; 53,9% de meninas) selecionados aleatoriamente nas escolas de São José dos Pinhais, PR. Foram analisados: sexo, estado nutricional, classe econômica e maturação sexual. Foram mensurados o nível de AF e o tempo de tela. As associações foram testadas com a regressão de Poisson e a fração atribuível populacional (FAP) verificou o impacto dos correlatos na presença combinada do nível insuficiente de AF e do elevado tempo de tela pela razão de prevalência (RP).

**Resultados:**

Foram considerados insuficientemente ativos 84,7% (n=666), com elevado tempo de tela, 96,4% (n=758), e 82,1% (n=645) dos adolescentes apresentaram a presença combinada desses comportamentos. O sexo feminino e a classificação econômica elevada estiveram positivamente associados aos níveis insuficientes de AF (RP=1,19; intervalo de confiança de 95% [IC95%] 1,12-1,27; FAP=15,97 - feminino/ajustado; RP=1,1; IC95% 1,01-1,19; FAP=9,09 - classe elevada/ajustado). O primeiro também se apresentou positivamente associado ao elevado tempo de tela após os ajustes (RP=1,18; IC95% 1,10-1,27; FAP=15,25) e mostrou-se associado positivamente à presença combinada desses comportamentos (RP=1,18; IC95% 1,10-1,27), com um impacto de 15,25% sobre essas condutas.

**Conclusões:**

Correlatos da AF podem ter impacto sobre o nível insuficiente de AFs e elevado tempo de tela, quando investigados de formas isolada e combinada, principalmente em relação às meninas.

## INTRODUCTION

Physical activity (PA) is a complex behavior, influenced by several aspects.^1^ ^,^ ^2^ In the past decade, there has been increasing interest in identifying variables that could explain the adhesion and maintenance of PA in order to promote more specific and effective interventions, which contemplate multiple levels of influence.^1^ ^,^ ^2^ In this context, the correlates of PA, such as sex, nutritional status, economic class and biological maturation, have been widely analyzed, and can explain the variations in levels of PA among adolescents.^1^ ^,^ ^3^

Several factors affect the practice of PA, especially outdoors, such as the increasing progress of urbanization, the reduction of public spaces for this type of activity, the increasing numbers of violence, technological dependence and the many facilities obtained by modernization.^4^ These changes have caused the transition of PAs that are mostly performed in outdoor environments to indoor activities, which appear to be safer. On the other hand, activities performed in the household may lead to more sedentary lifestyles, and excessive screen time.^5^ Sedentary behaviors and/or excessive screen time have been reported in studies involving adolescents - for example, the National Students’ Health Survey (PENSE) indicated that more than half of the adolescents in the 9th grade carry out sedentary activities for three hours or more, for example, watching television, using the computer, playing videogames or other activities in which they are sitting down.^6^

Understanding the correlates with the insufficient practice of PA and high screen time isolatedly (insufficient practice of PA or high screen time) and combined (insufficient practice of PA and high screen time) is an important step, once these studies have investigated these variables only separately.^7^ ^,^ ^8^ It is known that both behaviors have correlates in common and, isolatedly, present impacts on health, such as the increasing risk of developing chronic conditions and death.^9^ It is possible that the combination of both habits present higher damage regarding the impact on health, when compared to isolated analyses. But, up until now, the investigations found in the literature have been presented separately. Therefore, the objective of this study was to identify the impact of PA correlates with the isolated and combined presence of insufficient practice of PA and high screen time among adolescents in São José dos Pinhais, PR. The results of this study can contribute with the understanding of the impact that the correlates of PA have on the combined presence of insufficient levels of PA and high screen time, besides providing subsidies for planning and assessing interventions focusing on the promotion of PA^10^ for this age group.

## METHOD

This is a cross-sectional study, with a representative sample of adolescents enrolled in the public education network of São José dos Pinhais, Paraná, South Brazil. The middle-sized city is part of the metropolitan region of Curitiba, and is the sixth largest city in the state, with an estimated population of 302,759 residents. The study was in accordance with the research regulations involving human beings, from the National Health Council (Resolution n. 466/2012), and was approved by the Research Ethics Committee of Universidade Federal do Paraná (CAAE: 36759414.0.0000.0102).

The sampling calculation first considered an association of 1.4^11^ among low levels of PA and high screen time, 50% prevalence of insufficiently active,^3^ 95% confidence interval (95%CI) (α=0.05), 80% power (β=0.20), resulting in a minimum sample of 486 subjects, with a chance of properly rejecting the null hypothesis in 95%. However, with an addition of 30% for possible losses and refusals, the minimum necessary sample for the study was estimated in 632 subjects.

With the necessary n for the study, three stages were established for data collection, as follows:

1. Selection of all schools that offered high school in the morning.
2. Simple random choice of a class in each grade of high school.
3. Invitation to all students in the class to participate, voluntarily, of the study (n=850).

Data collection was performed in a coordinated manner, in the classroom, by previously trained evaluators.

In total, 850 adolescents were assessed; however, the ones who presented with physical and/or cognitive limitations, which could limit the practice of PA (informed by the student) were excluded (n=2), or those who reported pre-pubertal maturation state (n=26). Sampling loss was considered for adolescents who did not bring the informed consent form signed by the parents or tutors, who refused to participate in the study, or who were absent on the day of data collection (n=36). Therefore, the final sample of the study was composed of 786 subjects, which allows the identification of prevalence ratios (PRs) above 1.24 as risk, and below 0.78 as protective, in prevalence rates above 50% for those who are sufficiently active, with 80% power of chances of properly rejecting the null hypothesis.

The variables sex and age were self-reported by the adolescents and categorized in male and female, or, for age, treated continuously. The level of PA was estimated by a questionnaire that measured PA among adolescents, in its adapted version, validated for the Brazilian population.^12^ This questionnaire presents an intraclass correlation coeficient (ICC) of 0.88, Spearman correlation of 0.62 (p<0.001) and Kappa index of 0.59. The adolescents reported the weekly frequency and duration in the participation of 22 types of PA, with moderate to vigorous intensity in the past week. The activity score was calculated as the sum of the product of the weekly frequency times the volume, in minutes, spent in each activity. For the analysis, adolescents with weekly volume of PAs lower than 420 minutes were considered insufficiently active.^13^

Screen time was estimated by the Adolescent Sedentary Activity Questionnaire (ASAQ),^14^ in its version validated for the Brazilian population.^15^ However, in this study we only approached the alternatives referring to screen time in hours and/or minutes during a typical weekday and weekend (ICC=0.90; 95%CI 0.86-0.93). The ones considered with high screen time reported spending ≥2 hours a day in these activities.^16^

To assess nutritional status, first we measured total body mass, with a portable digital scale by PLENNA (Acqua model, São Paulo, Brazil), with a 100 g resolution. Then, height was measured with a metric tape attached to the wall, with 0.1 cm accuracy.^17^ With these data, it was possible to calculate the body mass index (BMI), based on the ratio between body mass and square height [body mass (kg)/height (m)^2^], and posterior classification of the adolescents according to the cutoff points proposed by Conde and Monteiro.^18^ For analytical purposes, the adolescents were divided into “eutrophic” and “excess weight” (overweight and obesity).

The economic class of the adolescents was assessed based on the number of domestic utensils in the household, presence of a maid and schooling of the person financially in charge of the household.^19^ For the analyses, this variable was classified in three categories: “low” (classes C and D), “intermediate” (classes B1 and B2), and “high” (classes A1 and A2).

The stage of sexual maturation was determined by a method proposed by Tanner,^20^ according to which the maturational stages are divided in 1 (pre-pubertal), 2, 3, 4 (pubertal) and 5 (post-pubertal). The classification in stages was self-evaluated by the adolescents, using the pubic pilosity analysis through images.^21^ ^,^ ^22^ However, this study only considered adolescents in the pubertal and post-pubertal stages.

The description of the sample was carried out by distribution of absolute and relative frequencies, and their possible differences were verified using the chi-squared test. To answer to the objectives proposed, difference Poisson regressions were used, with robust variance, in order to identify possible associations of sex, nutritional status, economic class and sexual maturation with the isolated and combined presence of insufficient level of PA and excessive sceen time. For the analysis of combined presence, a new variable was created, contemplating the presence of both conditions (level of PA below recommended^13^ and high screen time).^16^ The variables were inserted in the model by the forced entry method. For the factors associated with the isolated and combined presence of the insufficeintly active level and high screen time, the population attributed fraction (PAF) was calculated, based on the PR, using the equation PAF=PR-1/PR.^23^ All of the analyses were carried out using the Statistical Package for the Social Sciences for Windows (SPSS^®^), version 24.0, with a 5% significance level.

## RESULTS

**Figure 1:**
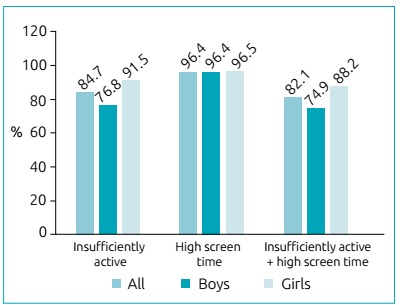
Isolated and combined presence of insufficient time of physical activities and high screen time among adolescents. São José dos Pinhais, PR, Brazil (n=786).

The final sample was composed of 786 adolescents (53.9% of girls), with mean age of 16.2±1.1 years. The highest proportion of adolescents presented with normal weight (82.7%), belonged to the intermediate economic class (57.6%), with pubertal maturational state (68.4%), insufficiently active (84.7%), with high screen time (96.4%), and combined presence of insufficient level of PA and high screen time (82.1%) (Table 1). Figure 1 presents the isolated and combined prevalence rates of risk behaviors in adolescents.

**Table 1:** Correlates and isolated and combined presence of insufficient level of physical activities and high screen time. São José dos Pinhais, PR, Brazil (n=786).

	Category	All		Boys		Girls		p-value
		n	%	n	%	n	%	
Sociodemographic aspects								
Sex	Male	-	-	362	46.1	-	-	
	Female	-	-	-	-	424	53.9	
Economic class	High	238	30.3	123	34	115	27.2	0.003**
	Intermediate	452	57.6	200	55.2	252	59.6	
	Low	95	12.1	39	10.8	56	13.2	
Biological aspects								
Nutritional status	Eutrophic	650	82.7	293	80.9	357	84.2	0.260
	Excessive weight	136	17.3	69	19.1	67	15.8	
Sexual maturation	Pubertal	538	68.4	214	59.1	324	74.4	0.001*
	Post-pubertal	248	31.6	148	40.9	100	23.6	
Isolated presence	Insufficiently active	666	84.7	278	76.8	388	91.5	0.001*
	High screen time	758	96.4	349	96.4	409	96.5	1.002
Combined presence	Insufficiently active +high screen time	645	82.1	271	74.9	374	88.2	0.001*

*p-values obtained using the chi-square test for correcting continuity; **p-values obtained using the chi-square test for linear tendency.

Table 1 presents the descriptive characteristics of the correlates and the isolated and combined presence of insufficient level of PA and high screen time. There were significant differences between genders for the economic class (p=0.003), sexual maturation (p=0.001), practice of PAs (p=0.01) and the combined presence of risk behaviors (p=0.001).

Table 2 shows the correlates associated with insufficient level of PAs in adolescents. Both in the crude and in the adjusted analyses, the female gender and the high economic classification were positively associated with insufficient levels of PA (PR=1.19; 95%CI 1.12-1.27; PAF=15.97 - female/adjusted; PR=1.1; 95%CI 1.01-1.19; PAF=9.09 - high class/adjusted). The female gender was also positively associated with high screen time (PR=1.18; 95%CI 1.10-1.27; PAF=15.25), and this association was verified only after the adjustments by the other variables in the model (Table 3).

**Table 2:** Correlates of physical activity associated to the insufficient level of physical activities in adolescents. São José dos Pinhais, PR, Brazil (n=786).

	Category	Insufficiently active									
		Crude analysis						Adjusted analysis			
		n	%	PR	95%CI	PAF (%)	p-value	PR	95%CI	PAF (%)	p-value
Sociodemographic aspects											
Sex	Male	423	53.9	1	-	-	-	1	-	-	-
	Female	362	46.1	1.15	1.10-1.21	13.04	0.001	1.19	1.12-1.27	15.97	0.001
Economic class	Low	95	12.1	1	-	-	-	-	-	-	-
	Intermediate	452	57.6	1.02	0.96-1.08	1.96	0.519	1.01	0.94-1.08	0.99	0.742
	High	238	30.3	1.10	1.03-1.19	9.09	0.005	1.10	1.01-1.19	9.09	0.016
Biological aspects											
Nutritional status	Eutrophic	136	17.3	1	-	-	-	1	-	-	-
	Excessive weight	649	82.7	1.04	0.98-1.10	3.85	0.172	1.06	0.99-1.13	5.66	0.094
Sexual maturation	Pubertal	538	65.8	1	-	-	-	-	-	-	-
	Post-pubertal	247	31.5	0.99	0.94-1.05	†	0.959	1.03	0.97-1.10	2.91	0.299

PR: prevalence ratio obtained from the Poisson regression with robust variance; 95%CI: 95% confidence interval; PAF; population attributable fraction. † PAF not calculated for PR lower than 1.0.

**Table 3:** Correlates of physical activity associated with high screen time among adolescents. São José dos Pinhais, PR, Brazil (n=786).

	Category	Screen time									
		Crude analysis						Adjusted analysis			
		n	%	PR	95%CI	PAF (%)	p-value	PR	95%CI	PAF (%)	p-value
Sociodemographic aspects											
Sex	Male	423	53.9	1	-	-	-	1	-	-	-
	Female	362	46.1	1.00	0.97-1.02	0.00	0.973	1.18	1.10-1.27	15.25	0.001
Economic class	Low	95	12.1	1	-	-	-	1	-	-	-
	Intermediate	452	57.6	1.01	0.98-1.04	0.99	0.472	1.01	0.94-1.09	0.99	0.472
	High	238	30.3	1.00	0.9-1.04	0.00	0.997	1.08	0.98-1.19	7.41	0.983
Biological aspects											
Nutritional status	Eutrophic	136	17.3	1	-	-	-	1	-	-	-
	Excessive weight	649	82.7	0.98	0.94-1.02	†	0.344	1.04	0.96-1.13	3.85	0.357
Sexual maturation	Pubertal	538	65.8	1	-	-	-	1	-	-	-
	Post-pubertal	247	31.5	1.01	0.99-1.04	0.99	0.201	1.04	0.97-1.12	3.85	0.224

PR: prevalence ratio obtained from the Poisson regression with robust variance; 95%CI: 95% confidence interval; PAF; population attributable fraction. † PAF not calculated for PR lower than 1.0.

Table 4 shows the correlates associated with the combined presence of insufficient level of PA and high screen time among adolescents. Regarding the analyses about the factors associated with the combined presence of risk behaviors, the crude analyses indicated associations with the female gender and the high economic class. When adjusted, only the female gender presented a positive association with the combined presence of risk behaviors (PR=1.18; 95%CI 1.10-1.27).

**Table 4:** Correlates of physical activity associated with the combined presence of insufficient level of physical activities and high screen time among adolescents. São José dos Pinhais, PR, Brazil (n=786).

	Category	Combined presence									
		Crude analysis						Adjusted analysis			
		n	%	PR	95%CI	PAF (%)	p-value	PR	95%CI	PAF (%)	p-value
Sociodemographic aspects											
Sex	Male	423	53.9	1	-	-	-	1	-	-	-
	Female	362	46.1	1.17	1.09-1.26	14.53	0.001	1.18	1.10-1.27	15.25	0.001
Economic class	Low	95	12.1	1	-	-	-	1	-	-	-
	Intermediate	452	57.6	1.02	0.94-1.10	1.96	0.525	1.01	0.94-1.09	0.99	0.714
	High	238	30.3	1.10	1.00-1.22	9.09	0.039	1.08	0.98-1.19	7.41	0.086
Biological aspects											
Nutritional status	Eutrophic	136	17.3	1	-	-	-	1	-	-	-
	Excessive weight	649	82.7	1.03	0.95-1.12	2.91	0.372	1.04	0.96-1.13	3.85	0.257
Sexual maturation	Pubertal	538	65.8	1	-	-	-	1	-	-	-
	Post-pubertal	247	31.5	1.01	0.94-1.08	0.99	0.783	1.04	0.97-1.12	3.85	0.227

PR: prevalence ratio obtained from the Poisson regression with robust variance; 95%CI: 95% confidence interval; PAF; population attributable fraction.

Based on the adjusted analyses about the associations between the correlates and the insufficient practice of PAs, the following was observed: for the female gender, PAF=15.97, and for the high economic class, PAF=9.09. Regarding screen time, the female gender presented PAF=15.25. In the analyses with the combined presence of risk behaviors, the female gender presented PAF=15.25.

## DISCUSSION

This study presents a measurement for the impact of PA correlates for its insufficient time and high screen time. The measurement of impact used was PAF, which enables to analyze more than measures of association; it allows to estimate the consequences and the repercussion of an exposure in relation to an outcome, as well as the proportion of new cases, which would not occur without exposure.^24^ In this study, PAF was estimated for the proportion of new cases of adolescents with the isolated presence of insufficient levels of PA, or high screen time, besides the combined presence of both behaviors. These facts have not been approached by the literature, which only analyzed, separately, PA correlates with PA level^3^ and screen time,^5^ ^,^ ^25^ also being limited to measurements of association.

The results in this study indicated positive associations of the female gender for the insufficient practice of PA (PR=1.19; 95%CI 1.12-1.27), for high screen time (PR=1.18; 95%CI 1.10-1.27) and for the combined presence of both behaviors (PR=1.18; 95%CI 1.10-1.27). The analyses of PAF indicate that, in relation to the boys, girls present 15.97% more chances of not fulfilling the recommendations for the practice of PA, 15.25% of presenting high screen time, beside the combined presence of both behaviors. It is clear that, during adolescence, the levels of PA decline^26^, whereas the time spent on sedentary activities increase,^27^ and these behavioral changes are mostly associated with the female gender.^26^ Also, other studies mention that girls most frequenty present the combination between insufficient level of PA and high screen time,^25^ which shows that these habits are more alarming for girls.^28^ Another explanation is that girls report receiving less social support from their families,^25^ besides the fact that, culturally, they are more encouraged to perform family activities in the household.^29^

These factors could explain the positive association observed among girls and the combined presence of the insufficient level of PAs and high screen time, showing the hypothesis that girls not only present with lower levels of PA in comparison to boys,^26^ ^,^ ^28^ but are also more prone to sedentary activities,^30^ and that is a matter of concern considering that risk behaviors adopted in adolescence may last until adulthood.^31^ Among adults, low levels of PA increase the chances of women having breast cancer in 10.1%, and in 9.4% the chances of men and women dying for different causes, according to the analyses of PAF and the findings by Lee et al.^32^

In this study, belonging to high economic classes was positively associated with the insufficient level of PA (PR=1.10; 95%CI 1.01-1.19), and these results are different from those found by Barbosa Filho,^3^ who found associations between economic class and excessive screen time, but not with insufficient levels of PA. The PAF analyses explain 9.09% of this situation, which seems to be clear for the literature,^33^ since similar analyses carried out in developed countries also observed that the low level of PA would increase the chances of children becoming obese in 11.4%.^34^ The fact is that even though higher economic and social conditions are apparently positively associated to lower levels of PA, this behavior is different between high and low-middle income countries,^33^ which has direct implications on the choices made by adolescents, and, consequently, requires different approaches in order to try and improve that scenario.^35^

Another point is that, in this study, most girls are pubertal (74.4%), and that phase is associated with growth spurt and changes in the body (increase in fat deposits) and in proportions (mammary development, hip amplification), besides the discomfort associated with regular menstrual cycles and reduced levels of hemoglobin in the blood. That may affect motor and physiological performance, and favor the adoption of more sedentary leisure activities,^36^ even though sexual maturation has not been associated with the isolated or combined presence of insufficient levels of PA and high screen time.

This study presented some limitations. One of them would be the use of self-declared measures to assess health behaviors. Even if widely used by epidemiological studies,^3^ ^,^ ^11^ self-declared methods present limitations as to their accuracy and tend to overestimate the answers. In this study, it was not possible to include adolescents from private schools, which partly restricts the extrapolation of data, indicating caution in the interpretation of the results. Another limitation is the fact that the study has a cross-sectional design, so it was not possible to establish and cause and effect relationship, which can lead to reverse causality.

This investigation presents strong aspects that must be highlighted. The study estimated the impact of PA correlates in the isolated and combined presence of low levels of PA and high screen time in a representative sample of student adolescents from São José dos Pinhais, PR. Another strong characteristic of the study is the sample size, which is adequate for the association analyses between the variables. Besides, the instruments used were previously tested and have adequate psychometric qualities for the application among students in the age group of interest. Further investigations could assess, in a combined manner, the impact that low levels of PA and high screen time can cause on the health of the population, as well as regarding the onset of diseases. Analyses that include psychosocial variables in risk behaviors can still show interesting results, as well as the inclusion of other behaviors that have a negative impact on health.

In conclusion, the findings indicate that PA correlates can have an impact on the insufficient level of Pas and high screen time, especially for girls. With these findings, the interventions and actions aiming at the promotion of PA should consider strategies to reduce screen time among girls and increase their participation in Pas. Another fact to be considered in actions to promote PA are adolescents from high economic classes.
